# Supplementary material for: State-switching navigation strategies in C. elegans are beneficial for chemotaxis
Source: ArXiv. 2025 Jul 31:arXiv:2508.00191v1. Preprint. [Version 1] (PMC12324552)
Supplement: Supplement 1 [file NIHPP2508.00191v1-supplement-1.pdf]

Supplementary information

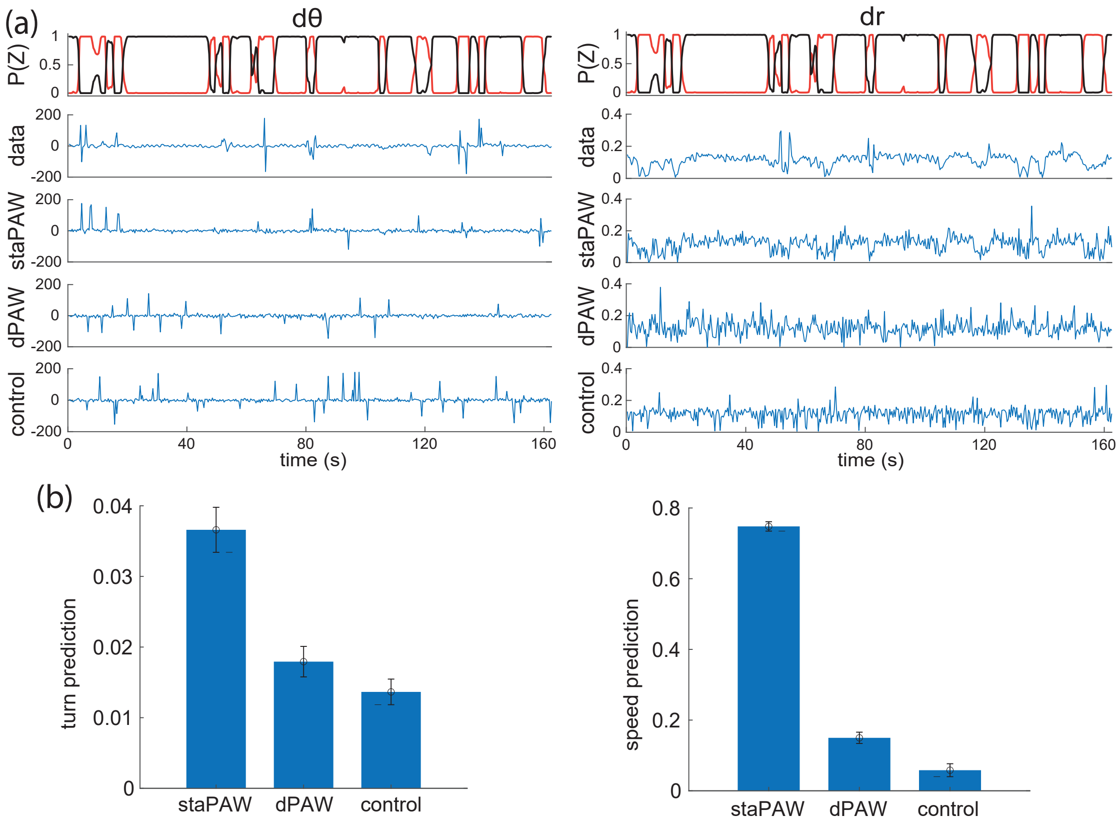

Supplementary Figure 1: The staPAW model better predicts time series of worm navigation behavior. **(a)** An example navigation time series with heading change (left) and speed (right) from experimental measurements (data) is shown in the second row. The posterior state probability and output of staPAW are shown in black (S-state) and red (T-state) lines. The output from dPAW and a control model without concentration input are shown below.**(b)** Across 500 sampled trajectories shown in (a), the prediction of turning is computed with Jaccard similarity index and prediction of speed is computed with correlation coefficient for three different models. Error bars show standard error of mean.

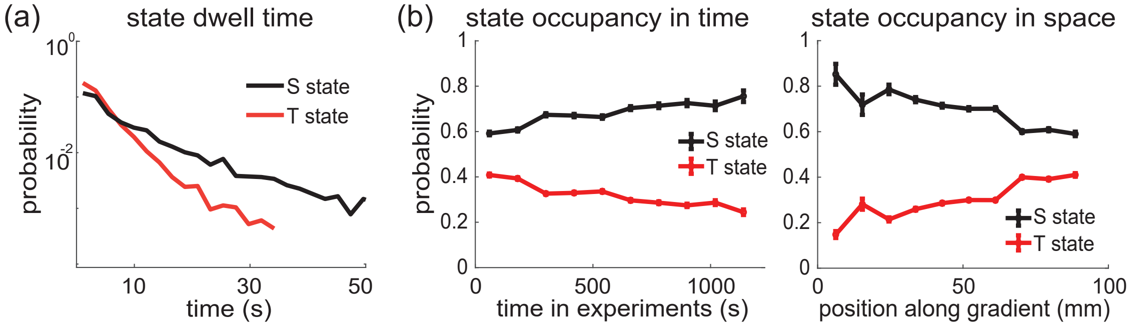

Supplementary Figure 2: Spatiotemporal distribution of states. **(a)** Dwell time distribution of two states in chemotaxis experiments. **(b)** The state occupancy across experimental time (left). Error bars show counting statistics of ~ 300 navigation tracks. The occupancy along the position of the linear salt gradient is shown on the right.

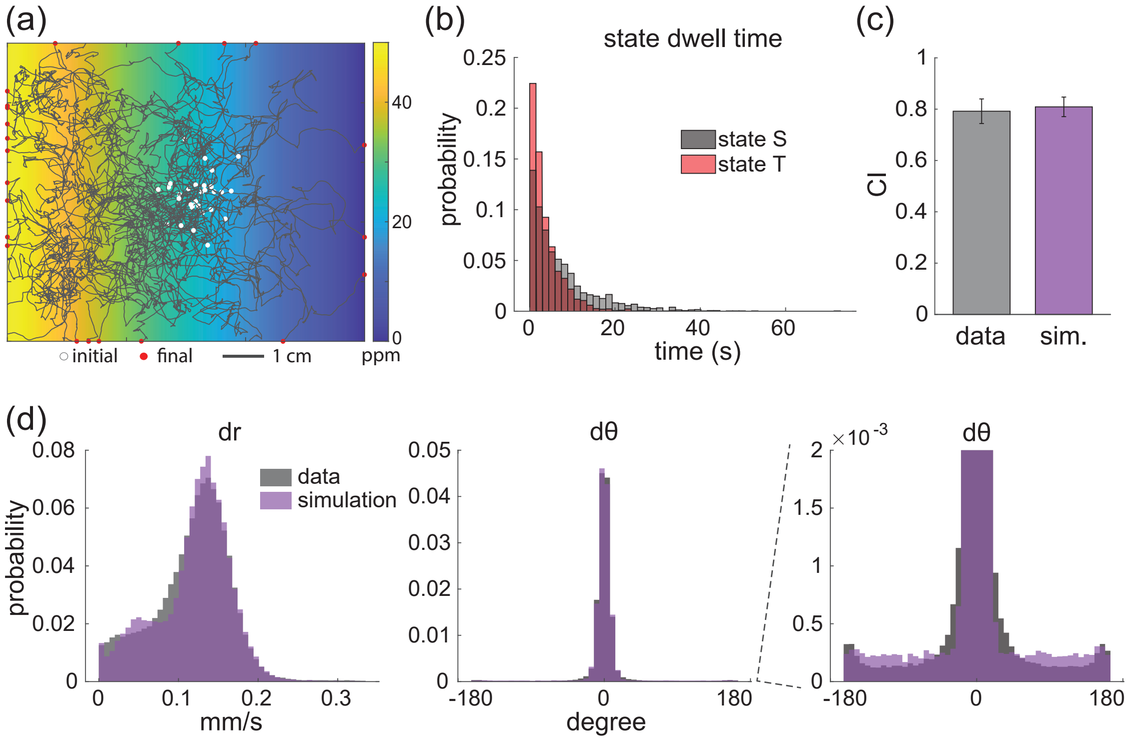

Supplementary Figure 3: The staPAW model generates navigation behavior similar to experimental observations. **(a)** Simulation of staPAW with parameters fitted to salt chemotaxis in the same environment. **(b)** The dwell time distribution of two states measured from the simulated trajectories in (a). **(c)** Chemotaxis index (CI) of the data and simulation. Error bars show standard deviation of 10 repeats of 50 sampled navigation trajectories. t-test shows no significant difference. **(d)** The distribution of speed dr and heading change $d\theta$ for experimental data and simulations from the fitted staPAW model.

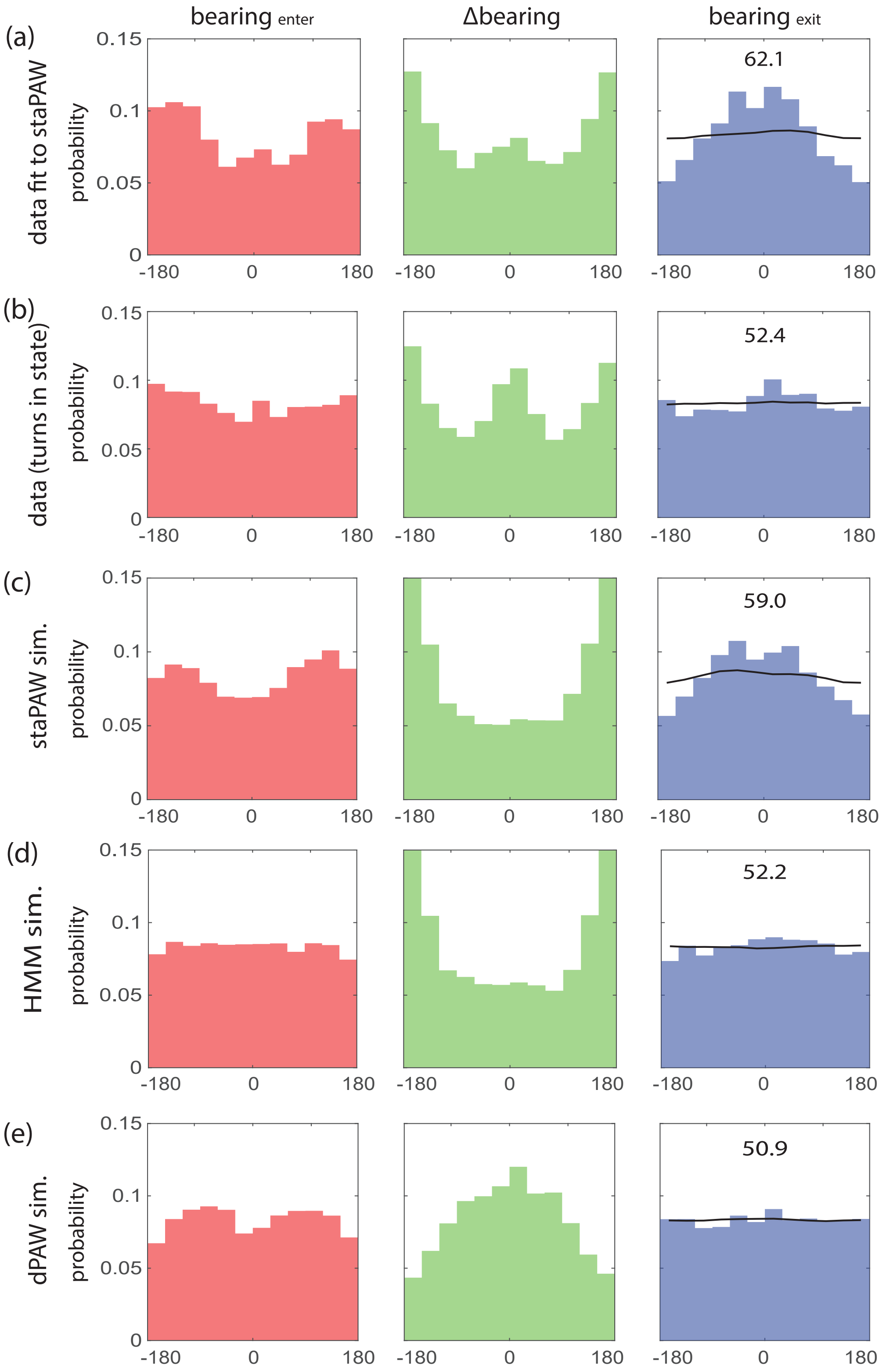

Supplementary Figure 4: Bearing angle around state transition for data and models. **(a)** The bearing angle before entering the T-state (left), the change in bearing angle in between (middle), and the bearing angle after exiting the turn (right). The bearing angle after shuffled Δbearing is shown in black line. The percentage of alignment to goal $P(|B_T| < 90)$ while existing events are shown in the right panels. The histograms are computed for T-states inferred in data. **(b)** Same as (a) but for individual turns within a pirouette state. **(c-e)** Same as (a) but for behavior simulated from models, including the full staPAW model (c), HMM that does not have sensory-driven states (d), and dPAW that does not have state-dependency (e).

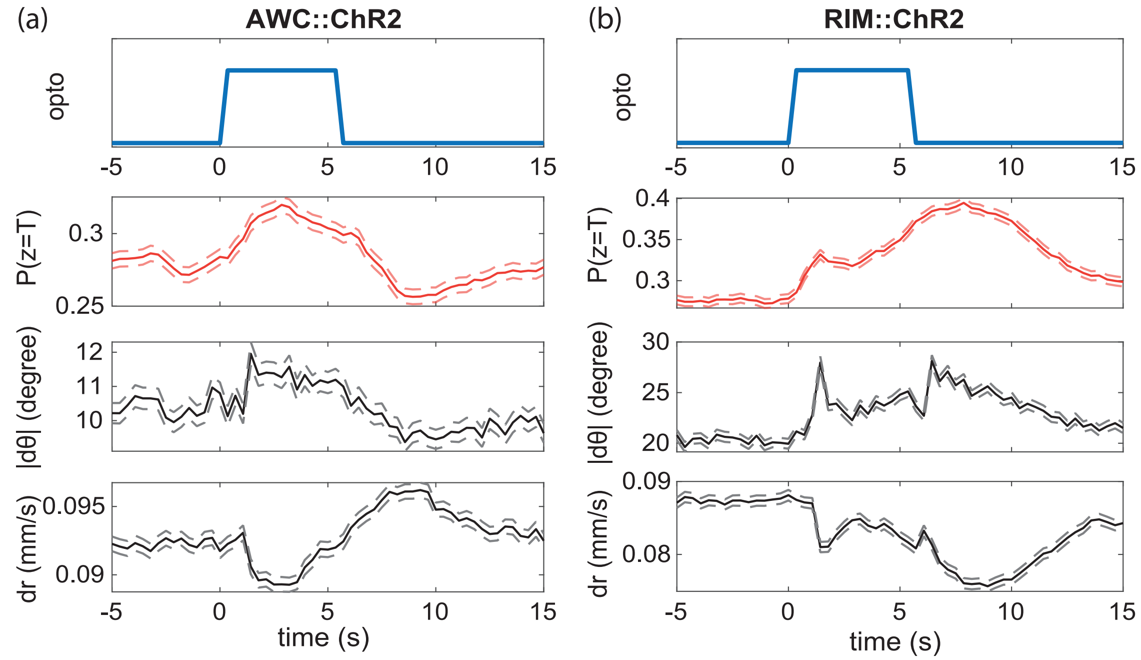

Supplementary Figure 5: The staPAW model captures state-dependent behavior driven by optogenetic input. **(a)** Behavioral response and the posterior probability of entering the turn-enriched state ($P(z = T)$) aligned to optogenetic input. The dash lines show standard error of mean. Measurements from 5000-6000 impulses are included. AWC::ChR2 strain is used for (a) and RIM::ChR2 is shown in **(b)**.

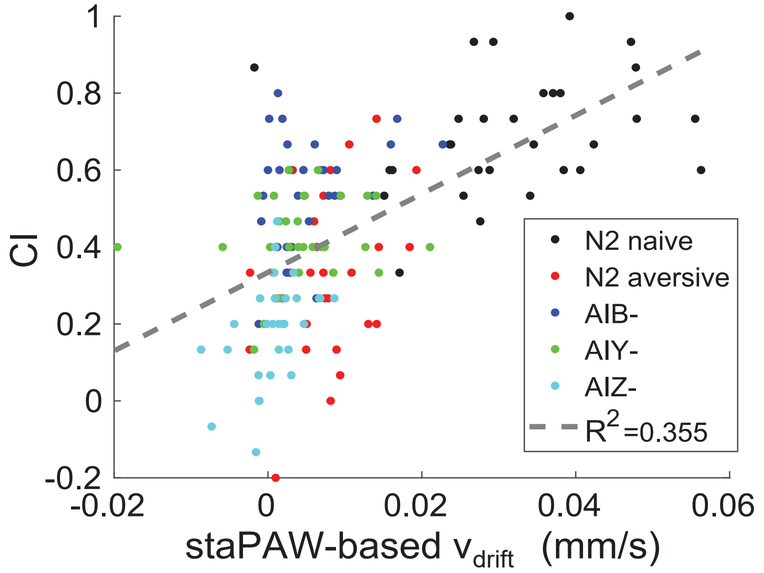

Supplementary Figure 6: Comparing model-predicted drift velocity (v_{drift}) and chemotaxis index (CI). Color code indicates different worm strains and conditions, each with 30 sub-sampled tracks, and dash line shows linear regression. The estimate drift is computed with $v_{\text{drift}} = \langle dr \rangle_S P(S)(P_{|B_T^{\text{exit}}|<90} - P_{|B_T^{\text{enter}}|<90})$, where $\langle dr \rangle_S$ is the average speed in S-state, $P(S)$ is the probability of being in S-state, and the last term is the difference between probability of aligned bearing exiting and entering T-state. This calculation simplifies the navigation problem into a Brownian ratchet, which describes how drift of a particle can result from diffusion across an asymmetric landscape [60, 61]. In the staPAW model, the asymmetry in movement direction mainly arise from the difference going up or down gradient around the T-state and average speed in the S-state.

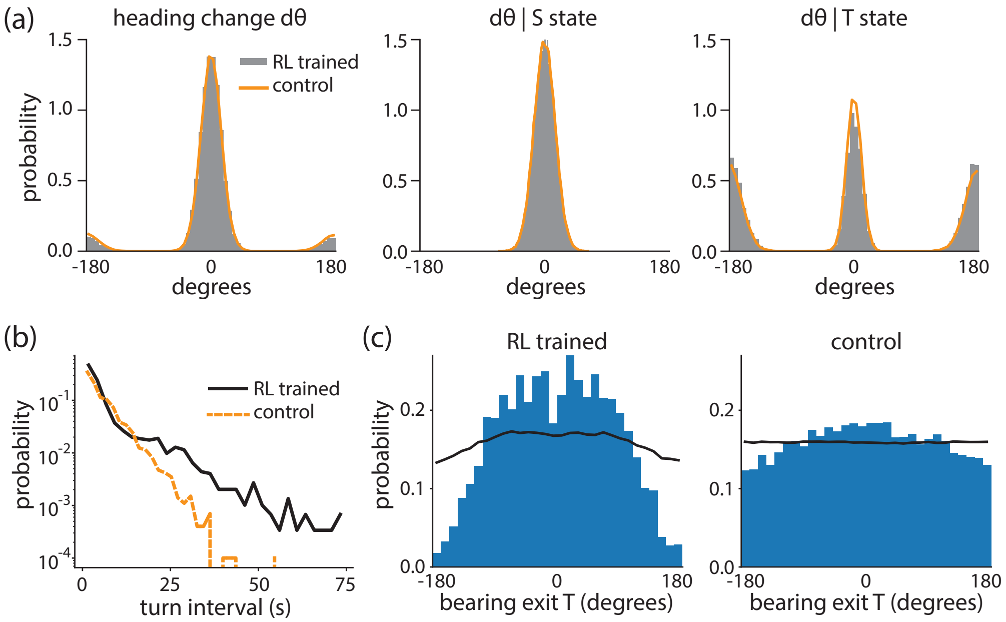

Supplementary Figure 7: Data-constrained RL model produces state-switching navigation strategies that qualitatively matches observations in worms. **(a)** The distribution of heading change $d\theta$ for all simulations (left), conditioned on S-state (middle), and conditioned on T-state (right), for both RL trained agents and a control model that does not have stimulus-dependent state transitions. **(b)** The turn interval distribution for RL trained model and control. **(c)** The distribution of bearing exiting T-state for RL trained (left) and control (right), with shuffled bearing differences shown in black line.
